# Task-based effective connectivity finds alterations in frontoparietal network in Duchenne muscular dystrophy

**DOI:** 10.1093/braincomms/fcaf356

**Published:** 2025-10-28

**Authors:** Mathula Thangarajh, Matthew Ridder, Hakinya Karra, Sanjana Javalkar, Edward Zuniga, Amy Harper, Nitai D Mukhopadhyay, Robert Cadrain, F Gerard Moeller, James M Bjork, Liangsuo Ma

**Affiliations:** Department of Neurology, Virginia Commonwealth University, Richmond, VA 23298-0599, USA; Stravitz-Sanyal Institute for Liver Disease & Metabolic Health, Virginia Commonwealth University, Richmond, VA 23298-0599, USA; Department of Neurology, Virginia Commonwealth University, Richmond, VA 23298-0599, USA; Department of Neurology, Virginia Commonwealth University, Richmond, VA 23298-0599, USA; Institute for Drug and Alcohol Studies, Virginia Commonwealth University, Richmond, VA 23298-0599, USA; Department of Neurology, Virginia Commonwealth University, Richmond, VA 23298-0599, USA; Department of Biostatistics, Virginia Commonwealth University, Richmond, VA 23298-0599, USA; Institute for Drug and Alcohol Studies, Virginia Commonwealth University, Richmond, VA 23298-0599, USA; Institute for Drug and Alcohol Studies, Virginia Commonwealth University, Richmond, VA 23298-0599, USA; Department of Psychiatry, Virginia Commonwealth University, Richmond, VA 23298-0599, USA; Institute for Drug and Alcohol Studies, Virginia Commonwealth University, Richmond, VA 23298-0599, USA; Department of Psychiatry, Virginia Commonwealth University, Richmond, VA 23298-0599, USA; Institute for Drug and Alcohol Studies, Virginia Commonwealth University, Richmond, VA 23298-0599, USA; Department of Psychiatry, Virginia Commonwealth University, Richmond, VA 23298-0599, USA

**Keywords:** effective connectivity, Duchenne muscular dystrophy, executive function challenges, functional diaschisis, perceptual processing

## Abstract

Duchenne muscular dystrophy is a monogenic X-linked genetic disorder that is caused due to the absence of dystrophin. In addition to the skeletal and cardiac manifestations, challenges in executive function are pervasive and persistent, affecting a majority of young individuals with Duchenne muscular dystrophy. Executive function-related disability is linked to chronic stress, academic under-achievement and poor vocational attainment. Of the executive function domains, inhibitory control and working memory are disproportionately affected, and linked to academic under-achievement in Duchenne muscular dystrophy. Despite its consequential importance to the quality-of-life in affected individuals, the neural substrates underpinning working memory challenges are poorly understood in this disease. The dynamic interactions of bilateral dorsolateral prefrontal cortex as part of the frontoparietal network is critical for working memory. Atypical neural connectivity within the frontoparietal network may underlie the neural basis of working memory challenges in Duchenne muscular dystrophy. Effective (directional) connectivity analysis of brain functional MRI is an advanced analytical approach that quantitates the directionality and the nature (facilitatory or inhibitory) causal interactions between brain regions. The strength of effective connectivity in Hertz—stronger (facilitatory) versus weaker (inhibitory)—within the frontoparietal network was analysed using dynamic causal modelling in 11 right-handed male participants with Duchenne muscular dystrophy and 9 right-handed male neurotypicals while they completed an *n*-back working memory task. Participants also completed standardized neurocognitive assessments out-of-scanner. Age-corrected working memory scores were comparable in Duchenne muscular dystrophy (mean 100.0, standard deviation 16.0) and neurotypicals (mean 109.0, standard deviation 8.0) (*P* = 0.15). Task-based hypoactivation of frontoparietal–occipital regions was observed in Duchenne muscular dystrophy. The group difference in mean frontoparietal effective connectivity during the in-scanner *n*-back working memory tasks was statistically lower by Bayes factor of 3 in Duchenne muscular dystrophy, compared to neurotypicals. The right posterior parietal → dorsolateral prefrontal connectivity correlated negatively to out-of-scanner working memory performance in Duchenne muscular dystrophy. Median reaction times during the 0-back and 2-back working memory tasks were longer in Duchenne muscular dystrophy compared to neurotypicals, but the difference did not reach statistical significance (*P* = 0.2). Median reaction time during the 0-back fearful facial condition was longer in Duchenne muscular dystrophy compared to neurotypicals (*P* = 0.01). Our work implicates atypical task-based effective connectivity within the frontoparietal network and impaired perceptual processing in Duchenne muscular dystrophy. Dynamic neural network signatures can serve as mechanistic targets for pharmacological and non-pharmacological interventions to mitigate executive function impairment in Duchenne muscular dystrophy.

## Teaser

Thangarajh *et al.* detected hypoactivation of bilateral frontoparietal brain regions during task-based functional MRI in Duchenne Muscular Dystrophy. Effective connectivity analysis revealed mutually inhibitory interactions between the right posterior parietal cortex and dorsolateral prefrontal cortex, supporting a functional diaschisis between perceptual processing and information encoding. Dystrophin-deficiency alters neural connectivity.

## Graphical Abstract

**Graphical Abstract:**
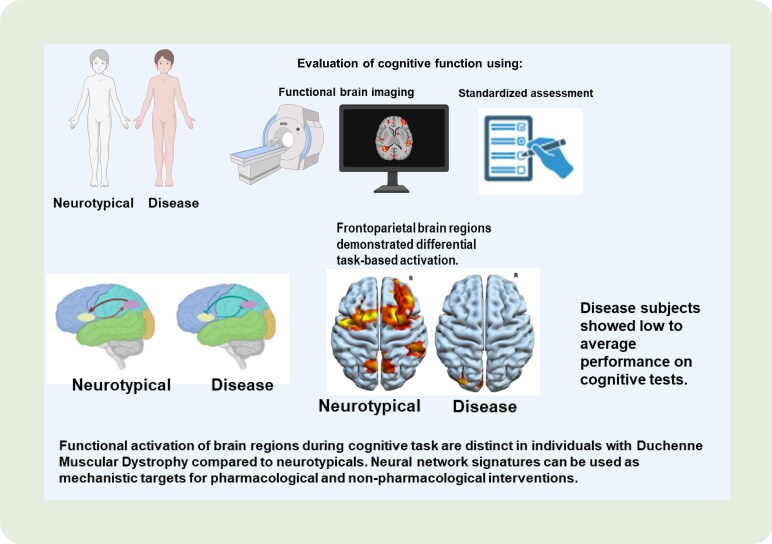

## Introduction

Duchenne muscular dystrophy (DMD) is the most common monogenic X-linked recessive genetic disease affecting boys worldwide, due to mutations in the *DMD* gene, resulting in the absence of dystrophin protein.^1^ Beyond skeletal pathology, there is a spectrum of cognitive abnormalities that are consequential for activities of daily living.^2^ An estimated 75% of boys experience cognitive symptoms, with approximately 50% meeting diagnostic criteria for neurodevelopmental or psychiatric illnesses.^3^ Particularly devasting are disabilities related to executive function (EF), namely—working memory, attention/inhibitory control and cognitive flexibility—critical for problem-solving, behavioural adaption and academic success.^4-7^ Despite these negative consequences, the neural substrates underlying EF deficits in DMD have not been investigated.

Brain imaging studies show both structural and functional abnormalities in DMD.^8-15^ Resting-state (task-negative) atypical functional connectivity within the ventromedial frontal cortex, posterior cingulate and adjacent praecuneus, has been described.^11^ Foundational for working memory is the activation and integration of bilateral dorsolateral prefrontal cortex (DLPFC), the anterior cingulate cortex (ACC) and the posterior parietal cortex (PPC), as part of the frontoparietal network (FPN)^16-21^ Among the FPN, prior neuroimaging work has shown that the DLPFC plays a decisive role in supporting working memory^19^ but relies on both the ACC^20^ and PPC^21^ for attentional control and perceptual information processing, respectively. Thus, atypical neural connectivity within FPN could underlie working memory deficits in DMD.

To gain neurobiological insights into FPN organization ‘during’ working memory, we analysed effectivity connectivity (EC) within FPN during an ‘in-scanner’ *n*-back working memory task. EC analysis is an advanced approach that provides quantitative estimates of directional influence of one neural population on another (i.e. how one brain region influences another).^22^ Unlike routine functional connectivity analysis which is based on temporal correlations of blood-oxygen-level-dependent (BOLD) signal among spatially distant brain regions,^23^ EC in dynamic causal modelling (DCM) is modelled at the latent neuronal level using a model of neural dynamics with a biophysically plausible forward model that describes the transformation from neural activity to measured BOLD signals.^24^

## Materials and methods

### Study participants and design

The study was conducted in full compliance with the Principles of Good Clinical Practice and was approved by the Institutional Review Board. Written informed consent was obtained from all parents; written assent from participants older than 12 years of age and verbal consent from participants less than 12 years of age were obtained prior to study commencement. A total of 20 right-handed participants underwent non-sedated, non-contrast brain MRI in this cross-sectional single-site study. Participants with DMD were between ages 8 and 14 years and had genetically confirmed out-of-frame *DMD* mutation. Ten of the 11 participants with DMD were on oral corticosteroid as standard-of-care. One DMD participant reported co-occurring attention-deficit hyperactivity disorder and autism spectrum disorder. None of the neurotypical controls (age range 8–16 years) had any medical or psychiatric diagnoses, nor were they on any psychotropic medications at study enrolment. All participants first underwent a mock scan during which they acclimatized in an open scanner for 15 min to alleviate potential procedural anxiety.

### Assessment of cognitive performance

Age-appropriate National Institutes of Health Toolbox (NIHTB)—Cognition Battery (NIHTB-CB), a validated psychometric measure to quantitate cognitive deficits in DMD, was administered using the iPad-application by a trained study team member.^25^ The NIHTB-CB assesses both fluid cognitive skills and crystalized cognitive skills, and then provides composite scores for fluid, crystalized and total cognitive abilities. The Total Cognition score is a normalized average of fluid and crystalized Cognition scores. Evaluation of EF [Dimensional Change Card Sort (DCCS)], episodic memory [Picture Sequence Memory (PSM)], working memory [List Sorting Working Memory (LSWM)], processing speed [Pattern Comparison (PCPS)] and attention (Flanker Inhibitory Control and Attention) summates Fluid Cognition. Assessment of reading and vocabulary includes Crystalized Cognition. Age-corrected standard scores (mean 100.0, standard deviation 15.0) of Total Cognition, Crystalized and Fluid Cognition scores were obtained and analysed as previously described.^25^

### Structural image acquisition

MRI data were acquired using a 3.0 Tesla Phillips Ingenia (Best, Netherlands) wide-bore scanner with 32-channel head coil. The scanning session began with a T1-weighted localizer scan (32 s) followed by a high resolution T1-weighted 3-Dimensional Magnetization Prepared Rapid Gradient Echo Structural Scan with acquisition voxel size of 1 × 1 × 1 mm and 160 axial slices (5 min and 38 s). Next, a T2-weighted Fluid-Attenuated Inversion Recovery (FLAIR) scan with voxel size of 1 × 1 × 5 mm and 28 axial slices (3 min and 9 s) was acquired to screen for incidental pathology.

### Functional image acquisition

Two runs of task-based functional magnetic resonance imaging (fMRI) (4 min and 57 s per run) were collected using gradient-echo single-shot echoplanar imaging, with repetition time = 1625 ms, echo time (TE) = 30 ms, flip angle = 52 degrees, field of view = [240 × 240]  mm, acquisition matrix = 96 × 96, in-plane voxel size = [2.5 × 2.5]  mm, slice thickness = 2.5 mm, interslice gap = 0.3 mm, 45 axial slices, multiband factor = 3, in-plane SENSE reduction = 1.5, 182 dynamic acquisitions.^26^

### *n*-Back task for fMRI

The *n*-back task is an ecologically valid assessment of working memory during which the study participant indicates with a button press when perceiving a stimulus that matches the one presented *n*-steps earlier.^27^ The *n*-back task was presented using E-Prime Professional software and utilized a block design for maximal power for contrasts between 2-back versus 0-back conditions (eight blocks each). Each picture was presented for 2 s followed immediately by a 1 s presentation of a fixation cross. The two-task conditions investigate facial recognition and emotional processes (faces) and working memory (places), respectively. Each of two *n*-back task runs was composed of eight trial blocks alternating with four 15 s periods of fixation cross. Each block began with either the 2-back or the 0-back instruction screen for 2.5 s, followed by 10 trials lasting 2.5 s. In each block of 10 trials, two stimuli were targets, 2–3 stimuli were non-target lures, and the rest were non-lures. Non-lures stimuli are only presented once in any given block. In total, there were 160 trials with 96 unique stimuli of four different stimulus types (24 unique stimuli per type). Human faces (either happy, fearful or neutral facial expressions) with facial expression stimulus type held constant within each block consisted of three-quarters of the stimuli types. The fourth stimulus type was composed of images of places (exterior scenes). For behavioural analysis, hit rates and false alarm (commission error) rates were *z* transformed, then D′ (sensitivity/accuracy) was calculated as the *z*-transformed hit rate minus the *z*-transformed false alarm rate, as previously described.^28^ Peripheral pulse and respirations were recorded during resting state and task-based fMRI for offline removal of aliased physiological signals.

### Pre-processing of fMRI

The fMRI images were minimally pre-processed using the default minimal pre-processing pipeline available in CONN 20B,^29,30^ which used both fMRI and structural images as inputs. The pipeline includes several steps, from functional realignment and unwarping to co-register all scans to the first scan, slice-timing correction to account for acquisition time differences among slices, identification of outliers by detecting individual scans with suprathreshold framewise displacement and/or global signal change values, direct segmentation and normalization to project functional images into the standard Montreal Neurological Institute 152 (MNI) reference space, resampling to 2 mm isotropic voxels, and application of functional smoothing using an 8 mm full width at half maximum Gaussian kernel. Because participants were children and boys, the tissue probability maps used for normalization were generated from CerebroMatic Toolbox^31^ specific to the age and male sex of the study participants. The CONN software works with Statistical Parametric Mapping (SPM) software (http://www.fil.ion.ucl.ac.uk/spm/) and Matlab (Mathworks Inc. Sherborn MA, USA) software. In this study, SPM12 (revision 7771) and Matlab 2020b were used together with CONN.

### SPM univariate analysis of contrast activation

The nodes of DCM were selected based on brain activation elicited by the working memory task using a two-step process, as described earlier.^32^ In the first-level univariate SPM analysis, two pre-processed fMRI runs were included in the model as two sessions. The 2-back and 0-back blocks were modelled by boxcar functions convolved with the SPM12 canonical hemodynamic response function. For each task condition (2-back and 0-back), parameters were estimated using General Linear Model^33^ without global normalization. Following the method described by Barch *et al*.,^34^ high-pass filter with a cut-off period of 200 s was applied for the fMRI data. Task-related brain activation was defined as the contrast of 2-back ‘minus’ 0-back parameters.

Next, an SPM12 second-level two-sample *t*-test was conducted voxel-wise for the 2-back ‘minus’ 0-back contrast image to detect between-group differences in regional activation. Under a cluster-defining height threshold (*P* = 0.001) and cluster extent size (*k* = 520),^35^ the statistical significance for between-group difference in brain activation was defined as family-wise error corrected cluster probability (*P*) < 0.05 (two-tailed).

An SPM12 second-level one-sample *t*-test was conducted to determine the DCM nodes, which were selected based on the regions that activated across both groups combined.^36^ Uncorrected cluster *P* value < 0.05 (two-tail)—commonly accepted^22^ for DCM node selection—was used as the activation threshold to define nodes for DCM analysis. For this second-level analysis, the cluster-defining threshold was *T* = 2.4. Anatomical labels for regions of activation were determined using the Anatomical Automatic Labelling 3 (AAL3) toolbox.^37^

### DCM

Task-based change in EC with DCM was performed, as described earlier.^32^ fMRI-based DCM is anchored on biophysical modelling of neuronal connectivity; the observed BOLD signal represents latent neuronal connectivity.^22^ We used the deterministic DCM in SPM12 software (Revision 7771; http://www.fil.ion.ucl.ac.uk/spm/) to measure EC, as described earlier.^23,32^ DCM is a system of bilinear differential state equations with coefficients measured in Hertz (Hz) units.^22,23^ A node in the biophysical model which receives the driving input is the brain region which first experiences change in neuronal activity. Subsequently, this node exerts influence on other interconnected nodes. Endogenous connectivity quantifies the strength (stronger or weaker) of EC between nodes and represents the inherent directionality and strength of connectivity regardless of momentary task conditions. Experimental conditions have the capability to modulate these inherent connectivities, quantifying their effects as increased or decreased connectivity strength in comparison to the baseline endogenous connectivity.

### Candidate node selection for DCM–EC analysis

Based on prior work demonstrating the role of FPN in working memory, we chose *a priori* candidate DCM nodes from this network.^38^ In addition, the anterior insula (INS) and putamen (PUT) were also selected as additional candidate DCM *a priori*, because in meta-analysis of brain regions activated by working memory tasks in children, the anterior INS and PUT also showed activation.^28,39^ The DCM nodes were specifically constrained by the task-related brain activation in two steps: First, the brain region corresponding to the candidate DCM node was required to show at least 10 active voxels as elicited by simple linear contrast, as detected during second-level SPM analyses. The threshold for the number of active voxels in defining the ROI for DCM is chosen to balance spatial specificity and signal-to-noise ratio. While the selection is somewhat arbitrary, it is typically set in intervals of 5 or 10 voxels. For instance, thresholds of 10, 20 and 50 voxels have been used previously.^40^ In this study, we adopted a threshold of 10 voxels, aligning with both existing conventions and our prior DCM research.^32^ Second, to precisely localize the position of each selected DCM node, the centre of each node was determined by the *t*-test maximum within the fMRI activation cluster corresponding to that node, and each selected DCM node had a spherical radius of 6 mm. In other words, *x*, *y*, *z* values (in mm) are the MNI coordinates of the centre of each node determined by the *t*-test maximum within the fMRI activation cluster corresponding to that node. Using this two-step procedure, we localized DCM nodes to the following structures and coordinates: right DLPFC (*x* = 42, *y* = 40, *z* = 30, *t* = 4.6), right ACC (*x* = 14, *y* = 20, *z* = 28, *t* = 4.9), left PPC (*x* = −52, *y* = 42, *z* = 46, *t* = 3.9), right PPC (*x* = 46, *y* = −40, *z* = 46, *t* = 4.3), right INS (*x* = 32, *y* = 18, *z* = 6, *t* = 3.7), right PUT (*x* = 28, *y* = 8, *z* = 0, *t* = 3.7), and left M1 (*x* = −30, *y* = −4, *z* = 54, *t* = 4.2). The fMRI time series for each node, which represents the temporal BOLD signal for that node, was obtained using the principal eigenvariate of that node.^41^ Additionally, based on the F-contrast of the effects of interest, each fMRI time series was adjusted as described previously.^41^

### Driving and modulatory DCM inputs to assess change in EC during *n*-back working memory task

Based on two types of task blocks, i.e. 2-back and 0-back blocks, two parametric regressors, called ‘All-Stimuli’ and ‘2-back-minus-0-back’, respectively, were created for the DCM analyses. The All-Stimuli regressor was all 2-back blocks and all 0-back blocks relative to the implicit baseline, and the 2-back-minus-0-back regressor was the 2-back blocks minus the 0-back blocks. In other words, the first regressor was non-specific visual stimulus effects, relative to the implicit baseline, while the second regressor modelled the added working memory demands of 2-back blocks over 0-back blocks. The All-Stimuli regressor was used as a single driving input to all the nodes of the DCM, and the 2-back-minus-0-back regressor was used as a putative modulator (modulatory input) of all EC. The modulatory input is the experimental factor ostensibly eliciting change in EC. This change in EC is inferred here as ‘working memory load’ modulation effects. For the sake of simplicity, each EC described in the rest of this paper was the working memory-driven modulatory change in EC triggered by the 2-back-minus-0-back modulatory input, in Hz.^42^

### Group-level EC analysis

After specifying an initial fully connected model (i.e. there were two bidirectional endogenous connectivities between any two nodes, the driving input affected all the DCM nodes, and the modulatory input affected all the endogenous connectivities), the Parametric Empirical Bayes (PEB) analysis,^43^ as implemented in DCM for SPM12 (Revision 7771), was used to conduct DCM group level analyses for the EC parameters. When PEB is used for comparing group difference in EC, there are two approaches: (i) compare the full PEB model to nested PEB models with certain parameters switched off (fixed at their prior mean of zero) to test specific hypotheses, and (ii) search over nested PEB models to simply prune away any parameters from the PEB which do not contribute to the model evidence. In this study, we selected the second approach, searching over nested PEB models, because it allows for a data-driven refinement of the model by pruning parameters that do not contribute to model evidence. This method is more flexible and avoids assumptions about specific hypotheses. By systematically removing unnecessary parameters, we ensure that the final model retains only the most meaningful effects. Three PEB analyses were conducted: (i) testing the mean of each modulatory change across all neurotypicals, (ii) testing the group difference in each modulatory change between DMD and neurotypicals and (iii) testing the relationship between each modulatory change and the NIHTB Fluid Cognition subscore using linear regression analysis in all participants. The PEB framework's linear regression analysis offers the advantage of automatically accounting for the covariance among all DCM parameters in the model. In this analysis, the beta (*β*) coefficient represents the slope of the linear relationship, indicating the extent of change in the outcome variable per unit of the predictor variable. For instance, a *β* value of 0.1 signifies a 0.1 Hz change in modulatory effects for each unit change in the NIHTB Fluid Cognition subscore.^25^

PEB employs Bayesian posterior inference,^22^ offering the advantage of minimizing false-positive results and alleviating the need to address the multiple-comparison problem.^22-24,32,44^ In PEB Bayesian posterior inference, the posterior probability (PP) indicates the degree of confidence in whether a modulatory change in a group differs from zero (or differs from another group), or the degree of the linear relationship between variables. The PP (0 ≤ PP ≤ 1) is the conditional probability computed by PEB using Bayes’ rule; it considers prior information such as the likelihood and the prior probability density of the model's parameters.^43,45^ The higher the PP value, the greater the confidence. In this study, a PP of greater than 0.95 (corresponding to a Bayes-factor of 3) indicates a reliable group difference or linear relationship between variables.

### Statistical analysis

The NIHTB-CB Total, Crystalized, and Fluid Cognition Scores are analysed here as standardized scores, based on individual performance compared to the population-normed score. In children, scores are age-corrected (mean 100.0, SD 15.0). Higher scores on the NIHTB-CB represents better cognitive performance. Median reaction time values in the *n*-back were calculated for each participant in each trial condition separately, to minimize effects of individual outlier trials. Then, individual-participant median reaction time values were compared between the two groups using *t*-test. Statistical analysis was performed using R software^46^ and plots were generated utilizing the ggplot2 library.^47^

## Results

### Demographic characteristics

A total of 20 participants (11 DMD and 9 neurotypicals) were evaluated. The mean age of participants with DMD was 11.5 years (range 8–14 years), and the mean age of neurotypical participants was 12.2 years (range 8–16 years). Table 1 summarizes the demographics of the study participants.

**Table 1:** Demographic characteristics of study participants

Group	DMD (*n* = 11)	Neurotypicals (*n* = 9)	
**Age in years**			
Mean and range	11.5 (8–14)	12.2 years (9–16)	
**Race**			
White	11	White	5
		Biracial	3
		Asian	1
**Ethnicity**			
Not Hispanic	9	Not Hispanic	9
Hispanic	2	Hispanic	0
***DMD* mutation**			
Deletion	7		
Nonsense	3		
Other	1		
**Co-morbid conditions**			
Attention-deficit hyperactivity	1		
Autism spectrum disorder	1		
**Oral corticosteroid use**			
Deflazacort	10		
Steroid-naive	1		

### Cognitive performance as assessed using NIHTB-CB

**Figure 1:**
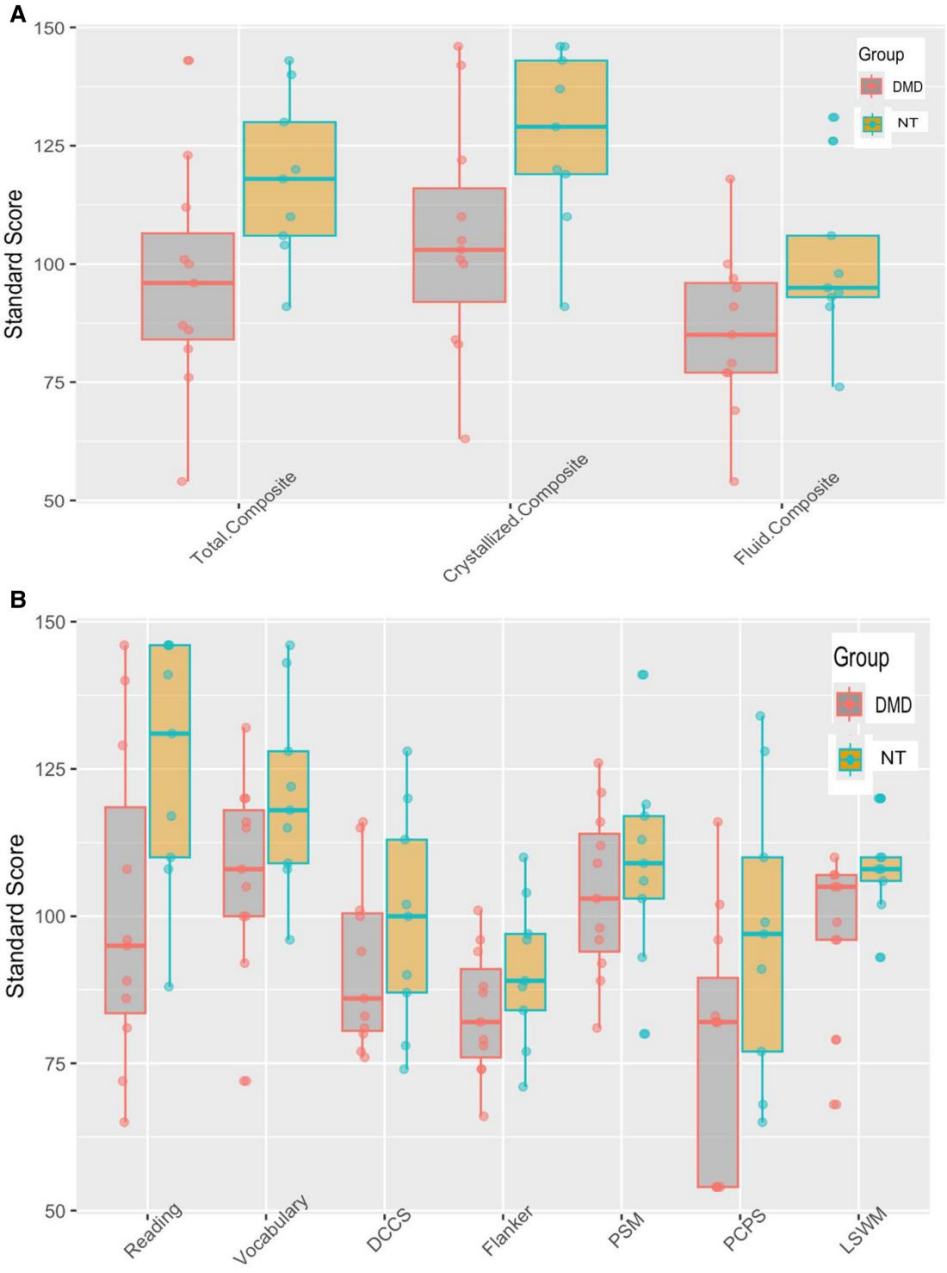
**Boxplots of the cognitive performance of study participants:** [11 male participants with DMD and 9 neurotypical (NT) males], as assessed by the NIHTB-CB. **A** depicts cognitive performance in the three domains of the NIHTB-CB, and **B** depicts cognitive performance on the subtests of crystalized and fluid cognition. The line inside the box represents the mean, and the ends of the boxes represent 25th and 75th percentiles of age-correct standard scores. The outlier values are indicated as dark dots. One-sample *t*-test with sample standard deviation (SD) was used; mean 100, SD ± 15 (*P* < 0.05). DCCS, dimensional change card Sort; PSM, picture sequence memory; LSWM, list sorting working memory; PCPS, pattern comparison; and Flanker, Flanker Inhibitory Control and Attention.

The NIHTB-CB Total Cognition score was significantly lower in participants with DMD (mean 96.4, SD 24.0; range 54.0–143.0) compared to neurotypicals (mean 118.0, SD 17.3; range 91.0–143.0) (*P* = 0.03). The NIHTB-CB Crystalized Cognition score was also significantly lower in participants with DMD (mean 105.0, SD 25.0; range 63.0–146.0) compared to neurotypicals (mean 127.0, SD 19.0; range 91.0–146.0) (*P* = 0.04). There was a trend for lower NIHTB-CB Fluid Cognition score in participants with DMD (mean 86.0, SD 17.0; range 54.0–118.0) compared to neurotypicals (mean 101.0, SD 18.0; range 74.0–131.0) (*P* = 0.07) (Fig. 1A). Both groups scored in the average range in working memory as assessed by the list sorting working memory task in the NIHTB-CB Fluid Cognition score (DMD: mean 100.0, SD 16.0; range 68.0–130.0; neurotypical: mean 109.0, SD 8.0; range 93.0–120.0) with no statistically significant group difference (*P* = 0.15) (Fig. 1B).

### Behavioural performance during *n*-back task

**Figure 2:**
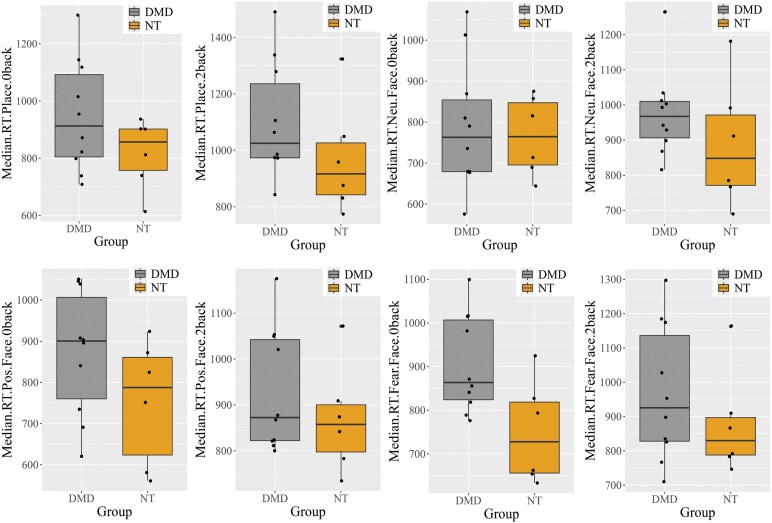
**Boxplots of the average median reaction time during the in-scanner *n*-back working memory (0-back and 2-back of places) and faces (facial recognition and emotional processes) task in the two groups of study participants (10 male participants with DMD and 6 neurotypical males).** The line inside the box represents the average of the median reaction time, and the ends of the boxes represent 25th and 75th percentiles of the distribution. The outlier values are indicated as dark dots. One-sample *t*-test was used for analysis, with statistical significance set at *P* < 0.05.

We compared reaction times during the in-scanner *n*-back task in study participants. The *n*-back task conditions included working memory (places) as well as facial recognition and emotional processes (positive, fearful, neutral faces). Three neurotypicals and one participant with DMD did not complete the in-scanner fMRI task and were not included in the analysis. There was no difference in the accuracy rates for either task condition between the two groups. The median reaction time values for places and faces were longer in participants with DMD than in neurotypicals. The median reaction time values for the 0-back and 2-back places conditions in participants with DMD (*n*  *=* 10) were marginally longer (*P* = 0.12) compared to neurotypicals (*n* = 6) (*P* = 0.2) (Fig. 2, top panel). Both groups took the least time to recognize neutral faces compared to positive or fearful faces (Table 2). Importantly, the reaction time during the 0-back of the fearful facial condition was statistically longer in participants with DMD compared to neurotypicals (*P* = 0.01). Although the other two facial recognition conditions and emotional processes (positive and neutral faces) were marginally longer in participants with DMD compared to neurotypicals, it did not reach statistical significance (Fig. 2; Table 2).

**Table 2:** Median reaction time in milliseconds of places and faces (facial recognition and emotional processes) in the two groups of study participants

	DMD (*n* = 10)	Neurotypicals (*n* = 6)	*P*-value
**Median RT positive face (2-back)**			
Mean (SD)	930 (133)	869 (117)	0.357
Median [Min, Max]	873 [801, 1180]	858 [735, 1070]	
**Median RT positive face (0-back)**			
Mean (SD)	873 (153)	752 (151)	0.152
Median [Min, Max]	901 [621, 1050]	788 [562, 923]	
**Median RT neutral face (2-back)**			
Mean (SD)	976 (123)	887 (180)	0.317
Median [Min, Max]	968 [816, 1270]	848 [690, 1180]	
**Median RT neutral face (0-back)**			
Mean (SD)	790 (156)	766 (96.2)	0.711
Median [Min, Max]	763 [576, 1070]	765 [645, 876]	
**Median RT fearful face (2-back)**			
Mean (SD)	967 (197)	877 (152)	0.323
Median [Min, Max]	926 [712, 1300]	830 [746, 1160]	
**Median RT fearful face (0-back)**			
Mean (SD)	906 (112)	749 (117)	0.025^a^
Median [Min, Max]	864 [776, 1100]	728 [634, 925]	
**Median RT place (2-back)**			
Mean (SD)	1100 (203)	969 (199)	0.222
Median [Min, Max]	1020 [843, 1490]	917 [774, 1320]	
**Median RT place (0-back)**			
Mean (SD)	947 (195)	818 (124)	0.127
Median [Min, Max]	913 [709, 1300]	857 [614, 937]	

^a^Statistically significant; RT, reaction time.

### Activation by the 2-back versus 0-back working memory contrast

**Figure 3:**
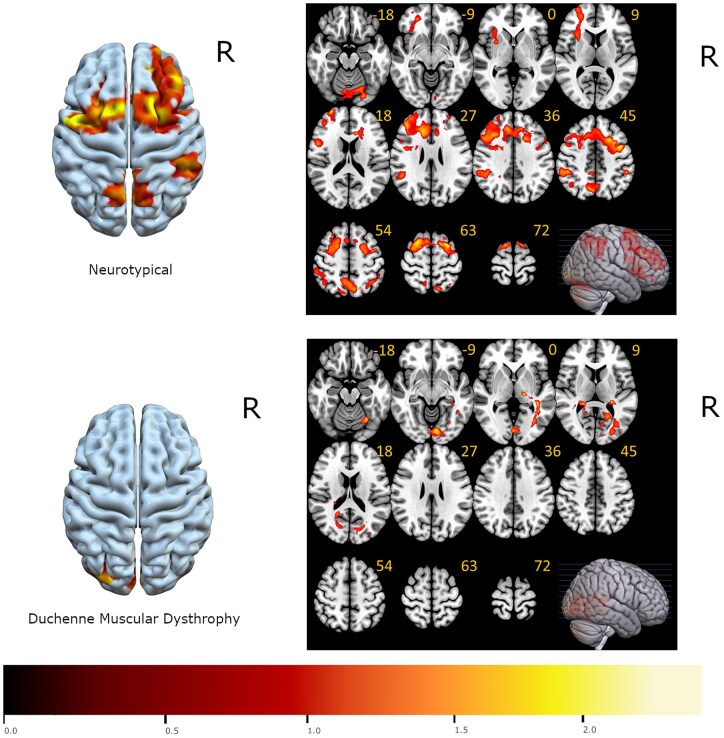
**Statistical comparison of FDR-corrected brain activations during the ‘in-scanner’ 2-back versus 0-back working memory task across the 10 male participants with DMD and 6 neurotypical males.** Brain activations are overlaid in colour on axial slices of MNI template brain, with the MNI *z* coordinate listed below each slice. The brain areas that are statistically different in activation between neurotypicals and participants with DMD are illustrated in colour; *t*-statistical map represents *t*-test at each voxel with statistical significance set at *P* < 0.05. The right (R) side of each slice is the right brain hemisphere.

We first localized brain regions activated during the *n*-back working memory task. The 2-back versus 0-back contrast elicited canonical recruitment of the dorsal attention network in neurotypicals, but not in DMD participants, resulting in a significant decrement in direct comparison of contrast activation between groups (Fig. 3).

### DCM–EC of the DLPFC within FPN during *n*-back working memory task

**Figure 4:**
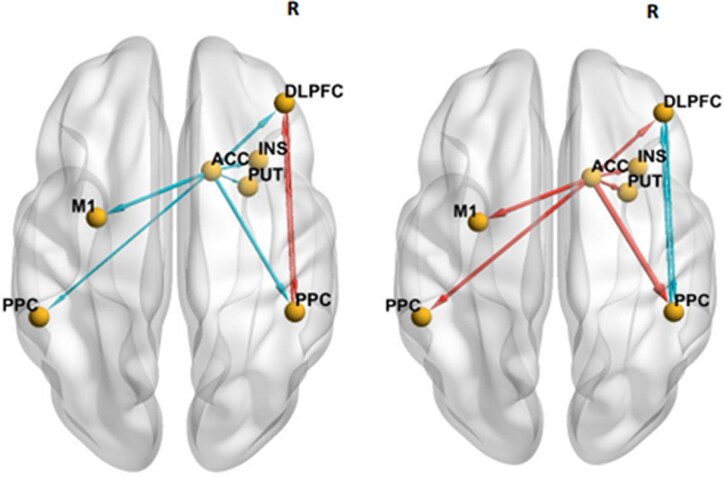
**Effective connectivities during the ‘in-scanner’ *n*-back task.** Lines with arrows representing effective connectivities during the ‘in-scanner’ *n*-back task in typically developing (left) and group difference in DMD (*n* = 10) relative to neurotypicals (*n* = 6) (right) as visualized with BrainNet viewer. The red line arrow denotes stronger effective connectivity and blue line arrow denotes weaker effective connectivity. The viewer’s right side of brain is the right (R) brain hemisphere. DLPFC, dorsolateral prefrontal cortex; PPC, posterior parietal cortex; INS, insula; PUT, putamen; ACC, anterior cingulate cortex; M1, primary motor cortex. Group-level effective connectivity was considered statistically significant if the posterior probability was greater than 0.95 using the PEB approach.

**Figure 5:**
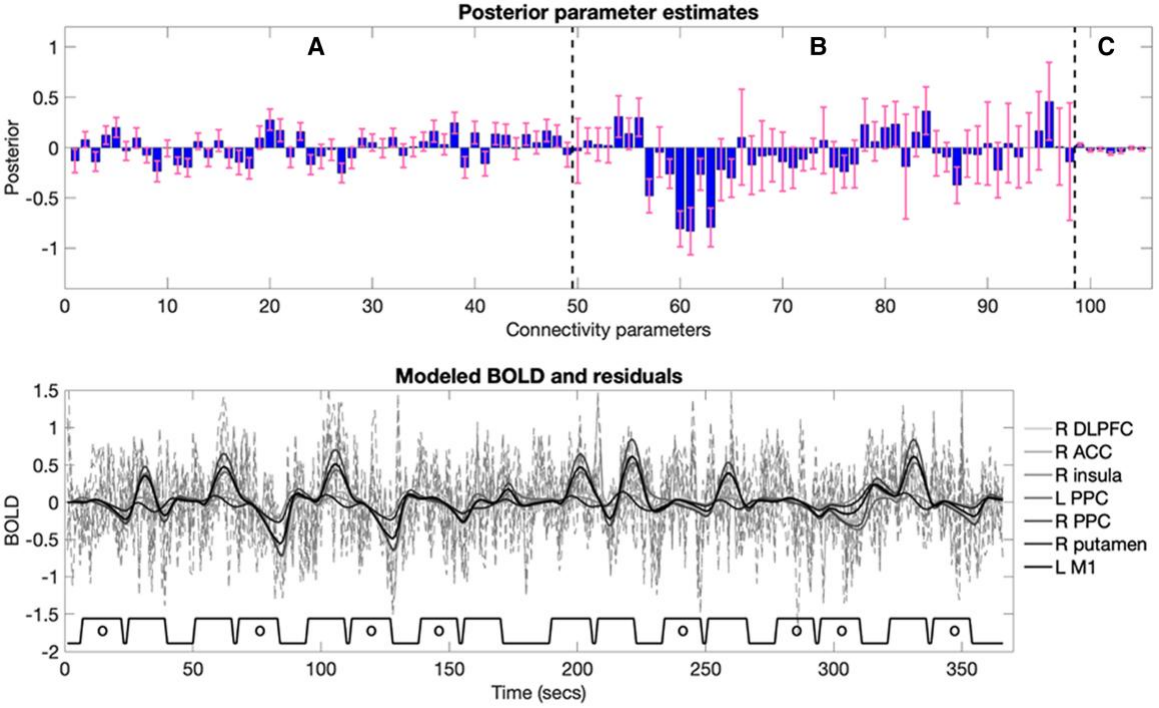
**Example DCM parameters and model fit for a randomly selected subject.** Top panel: Posterior DCM parameter estimates, with vertical dashed lines distinguishing different parameter types: **A** (endogenous connectivity), **B** (modulation effects of ‘working memory load’), and **C** (‘All-stimuli’ driving input). Error bars represent 90% credible intervals, derived from the posterior variance of each parameter. Bottom panel: Predicted timeseries for an example subject (solid lines), with each line representing a different brain region. Dashed lines indicate the model plus residuals. Below, blocks illustrate the timing of the 2-back (without circle ‘o’) and 0-back trials (with circle ‘o’). Figure format based on Zeidman *et al*.^42^

To understand the organization of the DLPFC within the FPN *during* working memory, we measured the DCM–EC during an *n*-back working memory task performed during fMRI. The ECs between the right DLPFC and right PPC were positive in neurotypicals (0.5 Hz) (Fig. 4, left) but by contrast was negative (−0.5 Hz) in participants with DMD (Fig. 4, right). Further, right DLPFC → right PPC and right PPC → right DLPFC interaction was mutually inhibitory (−0.5, −0.6 Hz, respectively). The group difference in DCM–EC demonstrated stronger neural connectivity between the right ACC and all other DCM nodes in participants with DMD, whereas DCM–EC was weaker in neurotypicals (Table 3). The right ACC → right PPC (1.2 Hz) and right ACC → right DLPFC (0.7 Hz) interactions was stronger in DMD but inhibitory in neurotypicals (−0.7, −0.6 Hz, respectively). Table 4 lists the posterior probability values of the driving input. As an example, the DCM parameters (top panel) and model fit (bottom panel) for a randomly selected subject is illustrated in Fig. 5.

**Table 3:** List of corresponding value for each effective connectivity in hertz during the in-scanner *n*-back task

Mean effective connectivity in the neurotypical group			Difference in the mean effective connectivity (Duchenne Muscular Dystrophy minus neurotypical group)	
Connection	Mean effective connectivity (Hz)	Posterior probability (PP)	Mean effective connectivity (Hz)	Posterior probability (PP)
Right DLPFC → Right PPC	0.4881	1	−0.5346	1
Right DLPFC → Right INS	0.1270	0.61	−0.1432	0.59
Right ACC → Right DLPFC	−0.6537	1	0.7327	1
Right ACC → Right INS	−0.4942	1	0.6077	1
Right ACC → Left PPC	−0.5109	1	0.8190	1
Right ACC → Right PPC	−0.7498	1	1.2074	1
Right ACC → Right PUT	−0.3076	1	0.6377	1
Right ACC → Left M1	−0.6567	1	0.7432	1
Right PPC → Right DLPFC	0.4980	1	−0.6391	1
Left PPC → Right ACC	−0.2104	0.94	0	0
Left PPC → Right INS	−0.0981	0.59	0	0
Right INS → Right PUT	0.1282	0.70	0	0

DLPFC, dorsolateral prefrontal cortex; ACC, anterior cingulate cortex; PPC, posterior parietal cortex; INS, insula; M1, primary motor cortex.

**Table 4:** The posterior probability values of the driving input

Neurotypical group			DMD minus neurotypical group	
Brain region	Mean (Hz)	Posterior probability (PP)	Mean (Hz)	Posterior probability (PP)
Right DLPFC	0.056	0.10	0.025	0.3
Right ACC	0	0	0	0
Left PPC	0	0	0	0.76
Right PPC	0.090	0.91	−0.026	0
Right INS	0.063	0.15	0	0
Right PUT	0	0	0	0
Left M1	0	0	0.130	0.98

DLPFC, dorsolateral prefrontal cortex; ACC, anterior cingulate cortex; PPC, posterior parietal cortex; INS, insula; M1, primary motor cortex.

### Relationships between *n*-back-elicited DCM–EC of the DLPFC within FPN and other out-of-scanner cognitive task performances

**Figure 6:**
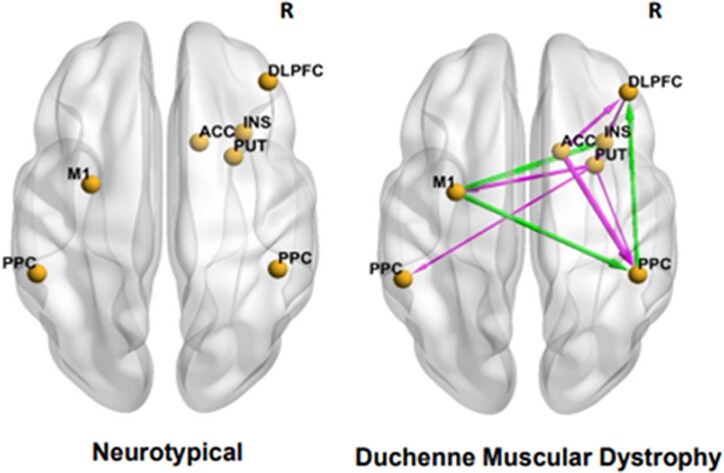
**Group-difference in task-based effective connectivities among frontoparietal nodes are discriminative of unique causal influences with out-of-scanner working memory performance.** Linear regression was used to correlate effective connectivity with age-adjusted NIHTB-CB list sorting working memory sub-scores in neurotypicals (*n* = 6) and in DMD (*n*  *=* 10). The pink line arrow denotes stronger EC whereas the green line arrow denotes weaker effective connectivity. The viewer’s right side of brain is the right (R) brain hemisphere. DLPFC, dorsolateral prefrontal cortex; PPC, posterior parietal cortex; INS, insula; PUT, putamen; ACC, anterior cingulate cortex; M1, primary motor cortex. A posterior probability greater than 0.95 using the PEB approach represents statistically significant linear relationship between the variables.

We wanted to evaluate whether ‘in-scanner’ working memory-driven DCM–EC as elicited by the *n*-back task generalized to similar task demands, as would be detected by a correlation with offline (out-of-scanner) working memory performance, as assessed with the list sorting working memory task of the NIHTB-CB Fluid Cognition score. To do so, we used the EC driven by the difference between the 2-back and 0-back conditions, and using linear regression calculated the correlation between DCM–EC of the DLPFC within FPN with the age-adjusted standard score of list sorting working memory task. In participants with DMD, the performance of the list sorting working memory task was negatively associated with the right PPC → right DLPFC EC (linear regression coefficient *β* −0.8), and positively associated with the right ACC → right DLPFC EC (linear regression coefficient *β* 0.7) and the right ACC → right PPC (linear regression coefficient *β* 1.2) (Fig. 6). None of the ECs showed linear relationship with the out-scanner working memory performance for the neurotypical group (Supplemental Table 1).

**Figure 7:**
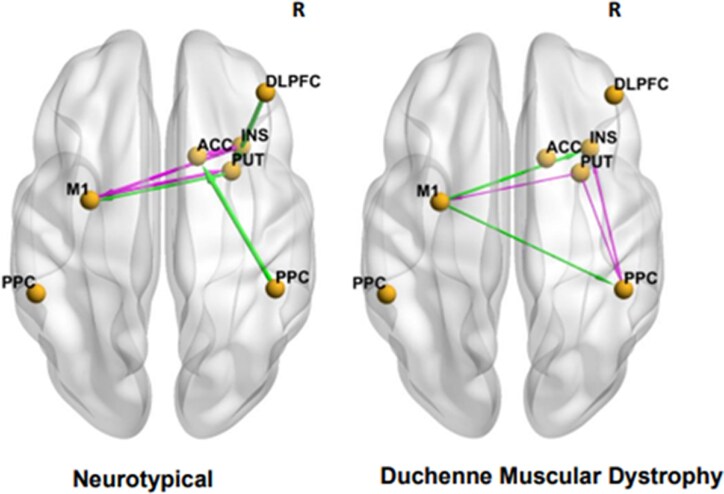
**Group-difference in task-based effective connectivities among frontoparietal nodes are discriminative of unique causal influences with out-of-scanner inhibitory control performance.** Linear regression was used to correlate effective connectivity with age-adjusted NIHTB-CB inhibitory control sub-scores in neurotypicals (*n* = 6) and in DMD (*n*  *=* 10). The pink line arrow denotes stronger EC whereas the green line arrow denotes weaker effective connectivity. The viewer’s right side of brain is the right (R) brain hemisphere. DLPFC, dorsolateral prefrontal cortex; PPC, posterior parietal cortex; INS, insula; PUT, putamen; ACC, anterior cingulate cortex; M1, primary motor cortex. A posterior probability greater than 0.95 using the PEB approach represents statistically significant linear relationship between the variables.

**Figure 8:**
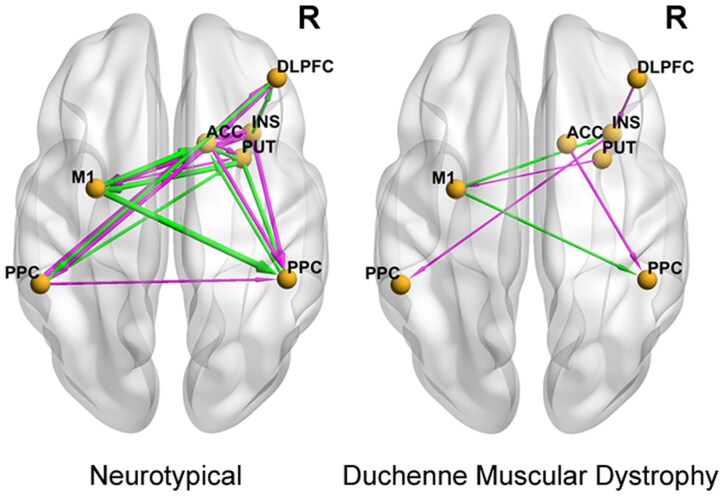
**Group-difference in task-based effective connectivities among frontoparietal nodes are discriminative of unique causal influences with out-of-scanner cognitive flexibility performance.** Linear regression was used to correlate effective connectivity with age-adjusted NIHTB-CB dimensional change card sort sub-scores in neurotypicals (*n* = 6) and in DMD (*n*  *=* 10). The pink line arrow denotes stronger EC whereas the green line arrow denotes weaker effective connectivity. The viewer’s right side of brain is the right (R) brain hemisphere. DLPFC, dorsolateral prefrontal cortex; PPC, posterior parietal cortex; INS, insula; PUT, putamen; ACC, anterior cingulate cortex; M1, primary motor cortex. A posterior probability greater than 0.95 using the PEB approach represents statistically significant linear relationship between the variables.

To test the specificity of DCM–EC for working memory demands versus inhibitory control and cognitive flexibility, we correlated DCM–EC of the DLPFC within FPN with performance on flanker inhibition/attention and dimensional change card sorting in the NIHTB-CB Fluid Cognition Score, respectively. Distinct EC patterns—including of the PUT and INS—were detected for inhibitory control and cognitive flexibility in DMD versus neurotypicals (Figs 7 and 8, respectively). There was stronger connectivity between the right PPC → right INS (*β* 0.4) and the right PUT → right PPC (*β* 0.3) in DMD participants (Fig. 7). Neurotypicals demonstrated more inter-hemispheric connectivity than DMD participants (Fig. 8). The EC between the right DLPFC → the right INS was stronger in participants with DMD (*β* 0.5). The individual *β* and posterior probability values for each connection within the FPN are listed below each panel (Supplemental Tables 2 and 3, respectively).

## Discussion

Second to skeletal muscles, dystrophin expression is second highest in the CNS.^48^ Human brain transcriptome data show that dystrophin transcripts are highly expressed in several cortical and subcortical brain regions.^49^ Postmortem analyses of DMD brains show that the lack of brain dystrophin leads to shorter dendritic length,^50^ fewer dendritic branching,^50^ astrogliosis,^50^ as well as synaptic proteomic changes.^51^ These morphofunctional changes may result in neural dysconnectivity in DMD. In line with such reasoning, resting-state hypoconnectivity in fronto-parietal regions has been described.^11^ Our study provides empirical support for neural dysconnectivity in DMD ‘during’ working memory, as evidenced by the global hypoactivation of several brain regions, including the FPN in DMD, compared to neurotypicals.

The dynamic interactions of the DLPFC with other brain regions within the FPN are critical for working memory performance.^52,53^ The DLPFC is rich in its connections with the parietal cortex, basal ganglia and thalamus. A hierarchical organization of executive control along a posterior-to-anterior axis is supported by the cascade model,^54^ with the PPC involved in perceptual information processing^55^ whereas the DLPFC supports information manipulation and action control.^56,57^ Our investigation into the EC of the DLPFC during an ‘in-scanner’ working memory task found atypical neural connectivity in DMD compared to neurotypicals, along the posterior-to-anterior axis. This finding suggests a functional diaschisis between the DLPFC and PPC during a working memory task in DMD, in that the dynamic communication between these two brain regions is not optimal for functional collaboration. Strong mutual inhibition may lead to diminished activation of these two brain regions, impairing the integration of visuospatial information and encoding of information for working memory. Indeed, the DLPFC and PPC were not significantly activated by the 2-back versus 0-back contrast in participants with DMD as shown in Fig. 3. The mutual inhibition of the DLPFC and PPC could disrupt information flow, and we put forth two possible explanations: the inhibitory influence of the PPC on the DLPFC may either prevent the information processed at the PPC (i) from being effectively transferred to, or (ii) being utilized by the DLPFC. Using Posner’s attention task, De Moura *et al*. observed that participants with DMD made more errors and had longer reaction times, highlighting the visuospatial attentional challenges in DMD.^5^ Our data corroborates this prior finding and suggests suboptimal PPC function as evidenced by the slower reaction times—representative of inefficient processing^58^—during the in-scanner working memory task (of up to 130 ms) in participants with DMD, although it did not reach a threshold for statistical difference.

The DLPFC shares extensive anatomical and functional connectivity with the ACC. Importantly, during a cognitively demanding task, the ACC moves from the role of an error monitor and becomes functionally integrated with the DLPFC.^59^ The ACC is an anatomical link between the DLPFC and the limbic areas, and in line with this role, the ACC is involved in both cognitive and affective processes.^60,61^ Our EC analysis detected stronger connectivity between the right ACC and other nodes within the FPN during the more arduous working memory demands. This finding would suggest that the ACC may provide compensatory support to the DLPFC. Projections of the pyramidal neurons within the ACC to regions of hypothalamus and periaqueductal grey matter mediate autonomic regulation;^61^ these control sympathetic responses such as an increase in heart rate during cognitive tasks. Interestingly, both poor heart rate variability as well as atypical galvanic skin response have been reported in DMD.^62^ Another interesting finding of our study was the longer reaction time for fearful facial recognition in participants with DMD, compared to neurotypicals. Unlike neurotypicals who recognized fearful facial expression in the shorter interval, participants with DMD took the longest during the 0-back to recognize fearful facial expression. Hinton *et al*. investigated four types of visual recognition and observed that participants with DMD performed statistically worse compared to siblings on facial affect.^63^ The fusiform gyrus of the temporal cortex and occipital brain regions are involved in facial recognition.^64^ Taken together, the alterations in autonomic control and facial affect described in DMD may be due to alterations in neural substrates due to brain dystrophin deficiency.

We also investigated the relationship between DCM–EC during an ‘in-scanner’ working memory task with out-of-scanner performance on working memory, inhibitory control and cognitive flexibility using NIHTB-CB. In line with evidence that within the FPN, there is further functional specialization of EF,^65,66^ we find distinct patterns of EC among the three FPN nodes for each cognitive task in DMD compared to neurotypicals. For example, inter-hemispheric connectivity was highly notable during tasks involving cognitive flexibility in neurotypicals but was lacking in DMD participants. Differences in functional connectivity in brain circuits that support cognitive flexibility has been shown to relate to differences in individual task performance.^67,68^ These patterns are in line with data from behavioural scales that reveal how many boys with DMD have real-world difficulty switching tasks and insist ‘on sameness and routines’.

Because it is modelled on latent neuronal fluctuations, EC provides biophysically informed insights on the dynamic information flow reflective of brain regional cytoarchitecture. Paquola *et al*. combined postmortem histology (Big Brain Atlas) and *in vivo* neuroimaging to study EC of the default mode network during resting state.^69^ They found distinct cytoarchitectural gradients in mesiotemporal and prefrontal regions that follow gradients of communication, varying from convergence of information flow in the mesiotemporal region, and an integration of information from disparate sources in the prefrontal region. Converging evidence from tracing experiments and recordings from non-human primates have demonstrated distinctive neuronal composition in the ACC versus DLPFC,^70,71^ as well as how the firing of specific neuronal populations is distinct during tasks involving spatial working memory.^72,73^ The ACC is rich in excitatory glutamatergic neurons that project to specific subpopulations of interneurons on the DLPFC.^74^ Specifically, calbindin interneurons from ACC disinhibit the activity of layer V pyramidal neurons in the DLPFC.^74^ Also, distinct groups of interneurons are involved in working memory^72^ and alterations in inhibitory interneuron subpopulations have been described in the *mdx* mouse model of DMD.^75^ Taken together, converging evidence from human and experimental models posit that dystrophin-deficiency likely alters neural network connectivity.

We acknowledge our study limitations. First, our sample size is relatively small as DMD is a rare genetic disease and inherently challenging to recruit study participants. Our methodological choice of DCM–EC ‘minimizes’ biases that may arise from conclusions using a small sample size. The age range of our study participants spanned late childhood and adolescence, both of which represent developmental epochs during which neural connectivity changes significantly. Future studies with pre-specified hypothesis can address how effective connectivity changes with age in DMD. Second, oral corticosteroids—the standard of care in DMD—has been shown to be associated with lower grey matter volume in DMD,^76^ and we cannot disentangle the effect of corticosteroid in our study. Third, fMRI-probed EF was limited to a working memory task for our experimental paradigm on feasibility and tolerability grounds, though neuropsychometric data indicate that inhibitory control and cognitive flexibility are also disproportionately affected in DMD.^25^ Also, our study participants had age-corrected scores in the average range, and we did not exclusively select participants with working memory deficits. Partly, we were constrained by availability of study participants, and study recruitment was based on convenience sampling. Last, we selected *a priori* DCM nodes previously reported to be important for executive functioning, and do not discount the possibility that other brain regions/nodes that modulate neural connectivity during EF.

Despite these limitations, we forecast our work to extend neural network signatures to be a pragmatic quantitative target for precision-medicine interventions in DMD. Brain functional connectivity studies in other neurobiological studies have documented the strong correlation between the dynamic configuration of neural circuits and clinical phenotype.^77-80^ Non-invasive transcranial magnetic stimulation (TMS) and neurofeedback are increasingly adopted in clinical practice; such techniques complement pharmacological as well as school-based resource facilitation, that can be facilitated towards academic outcomes in DMD. Esterman *et al*. used network-targeted TMS over the cerebellum and detected increased in network connectivity that paralleled an improvement in sustained attention as well as performance on neurobehavioral task.^81^ Such an approach will need dynamic neural network signatures that can serve as proxy-readouts of intervention; dynamic neural network signatures are superior compared to structural brain read-outs as well as intelligence quotient measures because both are relatively stable across developmental epochs. In sum, our proof-of-principle study provides preliminary empirical evidence of atypical neural connectivity during working memory demands in DMD.

## Supplementary Material

fcaf356_Supplementary_Data

## Data Availability

De-identified data will be shared upon reasonable request. A MATLAB implementation of the DCM approach is available as open-source code in the Statistical Parametric Mapping software package (https://www.fil.ion.ucl.ac.uk/spm/). The analytical code for NIHTB analysis is available at https://github.com/mthangarajh/Brain-Communications-.git
