# Correction to: Task-based effective connectivity finds alterations in frontoparietal network in Duchenne muscular dystrophy

**DOI:** 10.1093/braincomms/fcag118

**Published:** 2026-04-06

This is a correction to: Mathula Thangarajh, Matthew Ridder, Hakinya Karra, Sanjana Javalkar, Edward Zuniga, Amy Harper, Nitai D Mukhopadhyay, Robert Cadrain, F Gerard Moeller, James M Bjork, Liangsuo Ma, Task-based effective connectivity finds alterations in frontoparietal network in Duchenne muscular dystrophy, *Brain Communications*, Volume 7, Issue 5, 2025, https://doi.org/10.1093/braincomms/fcaf356

The following change has been made to the originally published paper.

The statement: “Open Access Publishing fee was supported by the American Neuromuscular Foundation.” has been added to the end of the **Funding** section within the paper.

