# Supplementary material for: Task-based effective connectivity finds alterations in frontoparietal network in Duchenne muscular dystrophy
Source: Brain Commun. 2025 Oct 28;7(5):fcaf356. doi: 10.1093/braincomms/fcaf356 (PMC12560160; doi:10.1093/braincomms/fcaf356)
Supplement: fcaf356_Supplementary_Data [file fcaf356_supplementary_data.docx]

**Supplementary Table 1. List of the beta (β) coefficient which represents the slope of the linear relationship in dynamic causal modeling endogenous parameter and modulatory estimates for all connections and their respective posterior probability.**

|  | Linear relationship between effective connectivity and working memory score in the typically developing group | | Linear relationship between effective connectivity and working memory score in the Duchenne Muscular Dystrophy group | |
| --- | --- | --- | --- | --- |
| Connection | Beta | Posterior probability (PP) | Beta | Posterior probability (PP) |
| Right DLPFC 🡪 Right DLPFC |  |  | -1.3 | 1 |
| Right DLPFC 🡪 Right INS |  |  | 0.56 | 1 |
| Right ACC 🡪 Right DLPFC |  |  | 0.78 | 1 |
| Right ACC 🡪 Right PPC |  |  | 1.24 | 1 |
| Right INS 🡪 Right INS |  |  | 0.62 | 1 |
| Right INS 🡪 Right PUT |  |  | -0.34 | 0.9 |
| Right INS 🡪 Left M1 |  |  | -0.37 | 1 |
| Left PPC 🡪 Left M1 |  |  | -0.12 | 0.59 |
| Right PPC 🡪 Right DLPFC |  |  | -0.85 | 1 |
| Right PUT 🡪 Right DLPFC |  |  | 0.20 | 0.7 |
| Right PUT 🡪 Right ACC |  |  |  |  |
| Right PUT 🡪 Right INS |  |  | 0.66 | 1 |
| Right PUT 🡪 Left PPC |  |  | 0.53 | 1 |
| Right PUT 🡪 Right PPC |  |  | 0.44 | 1 |
| Right PUT 🡪 Right PUT |  |  | -1.13 | 1 |
| Right PUT 🡪 Left M1 |  |  | 0.79 | 1 |
| Left M1 🡪 Right INS |  |  | -0.66 | 1 |
| Left M1 🡪 Left PPC |  |  | -0.25 | 0.69 |
| Left M1 🡪 Right PPC |  |  | -1.08 | 1 |

**DLPFC = Dorsolateral prefrontal cortex; ACC = Anterior cingulate cortex; PPC = Posterior parietal cortex; INS = Insula; M1 = Primary Motor Cortex.**

**Supplementary Table 2. List of the beta (β) coefficient which represents the slope of the linear relationship in dynamic causal modeling endogenous parameter and modulatory estimates for all connections and their respective posterior probability.**

|  | Linear relationship between effective connectivity and inhibitory control score in the typically developing group | | Linear relationship between effective connectivity and inhibitory control score in the Duchenne Muscular Dystrophy group | |
| --- | --- | --- | --- | --- |
| Connection | Beta | Posterior probability (PP) | Beta | Posterior probability (PP) |
| Right DLPFC 🡪 Right INS |  |  | 0.31 | 0.91 |
| Right DLPFC 🡪 Left PPC |  |  |  |  |
| Right DLPFC 🡪 Right PUT | -0.98 | 1 | -0.25 | 0.73 |
| Right DLPFC 🡪 Left M1 |  |  | -0.23 | 0.76 |
| Right ACC 🡪 Right PUT | 0.69 | 0.92 |  |  |
| Right INS 🡪 Right ACC | 0.85 | 1 |  |  |
| Right INS 🡪 Right INS |  |  | 0.65 | 1 |
| Right INS 🡪 Right PPC | 0.23 | 0.52 |  |  |
| Right INS 🡪 Left M1 | 0.68 | 1 |  |  |
| Left PPC 🡪 Right ACC | 0.69 | 0.88 |  |  |
| Right PPC 🡪 Right ACC | -0.95 | 1 |  |  |
| Right PPC 🡪 Right INS |  |  | 0.45 | 1 |
| Right PUT 🡪 Right PPC |  |  | 0.26 | 0.75 |
| Right PUT 🡪 Right PUT | -0.83 | 0.81 | 0.34 | 1 |
| Right PUT 🡪 Left M1 | -0.54 | 1 | -0.44 | 0.77 |
| Left M1 🡪 Right INS |  |  | 0.31 | 1 |
| Left M1 🡪 Left PPC |  |  | -0.56 | 1 |
| Left M1 🡪 Right PPC | -0.25 | 0.49 |  |  |
| Left M1 🡪 Right PUT | 0.67 | 1 | -0.46 | 1 |
| Left M1 🡪 Left M1 | 0.97 | 1 |  |  |

**DLPFC = Dorsolateral prefrontal cortex; ACC = Anterior cingulate cortex; PPC = Posterior parietal cortex; INS = Insula; M1 = Primary Motor Cortex.**

**Supplementary Table 3. List of the beta (β) coefficient which represents the slope of the linear relationship in dynamic causal modeling endogenous parameter and modulatory estimates for all connections and their respective posterior probability.**

|  | Linear relationship between effective connectivity and dimensional change card sorting score in the typically developing group | | Linear relationship between effective connectivity and dimensional change card sorting score in the Duchenne Muscular Dystrophy group | |
| --- | --- | --- | --- | --- |
| Connection | Beta | Posterior probability (PP) | Beta | Posterior probability (PP) |
| Right DLPFC 🡪 Right DLPFC |  |  |  |  |
| Right DLPFC 🡪 Right ACC | 0.72 | 1 |  |  |
| Right DLPFC 🡪 Right INS |  |  | 0.53 | 1 |
| Right DLPFC 🡪 Left PPC | -0.84 | 1 |  |  |
| Right DLPFC 🡪 Right PUT | -0.24 | 0.6 |  |  |
| Right DLPFC 🡪 Left M1 | 0.40 | 0.8 |  |  |
| Right ACC 🡪 Right DLPFC | 0.85 | 1 | 0.35 | 0.92 |
| Right ACC 🡪 Right INS | 0.68 | 1 | -0.16 | 0.56 |
| Right ACC 🡪 Left PPC | 0.92 | 1 |  |  |
| Right ACC 🡪 Right PPC | 0.80 | 1 | 0.58 | 1 |
| Right ACC 🡪 Right PUT | 0.68 | 1 |  |  |
| Right INS 🡪 Right DLPFC | 0.64 | 1 |  |  |
| Right INS 🡪 Right ACC | 0.91 | 1 |  |  |
| Right INS 🡪 Right INS | -0.23 | 0.45 | 0.29 | 0.7 |
| Right INS 🡪 Left PPC |  |  | 0.46 | 1 |
| Right INS 🡪 Right PPC | 0.90 | 1 |  |  |
| Right INS 🡪 Right PUT |  |  |  |  |
| Right INS 🡪 Left M1 | 1.30 | 1 |  |  |
| Left PPC 🡪 Right DLPFC | -0.31 | 0.63 |  |  |
| Left PPC 🡪 Right ACC | 1.07 | 1 |  |  |
| Left PPC 🡪 Left PPC |  |  | -0.54 | 1 |
| Left PPC 🡪 Right PPC | 0.50 | 1 |  |  |
| Left PPC 🡪 Right PUT | -0.63 | 1 |  |  |
| Left PPC 🡪 Left M1 |  |  |  |  |
| Right PPC 🡪 Right DLPFC |  |  |  |  |
| Right PPC 🡪 Right ACC | -0.66 | 1 |  |  |
| Right PPC 🡪 Right INS |  |  |  |  |
| Right PPC 🡪 Left PPC | 0.27 | 0.76 |  |  |
| Right PUT 🡪 Right DLPFC | -0.63 | 1 |  |  |
| Right PUT 🡪 Right ACC | -0.75 | 1 |  |  |
| Right PUT 🡪 Right INS | -0.47 | 1 | 0.30 | 1 |
| Right PUT 🡪 Left PPC |  |  |  |  |
| Right PUT 🡪 Right PPC | -0.89 | 1 |  |  |
| Right PUT 🡪 Right PUT | 0 | 0 | -0.26 | 0.61 |
| Right PUT 🡪 Left M1 | -1.16 | 1 | 0.32 | 1 |
| Left M1 🡪 Right DLPFC | -0.41 | 0.73 |  |  |
| Left M1 🡪 Right ACC | -1.13 | 1 |  |  |
| Left M1 🡪 Right INS |  |  | -0.53 | 1 |
| Left M1 🡪 Left PPC |  |  | -0.27 | 0.73 |
| Left M1 🡪 Right PPC | -1.35 | 1 | -0.566 | 1 |
| Left M1 🡪 Right PUT | 0.48 | 1 |  |  |
| Left M1 🡪 Left M1 | 2.36 | 1 |  |  |

**DLPFC = Dorsolateral prefrontal cortex; ACC = Anterior cingulate cortex; PPC = Posterior parietal cortex; INS = Insula; M1 = Primary Motor Cortex.**
